# An Ontology to Bridge the Clinical Management of Patients and Public Health Responses for Strengthening Infectious Disease Surveillance: Design Science Study

**DOI:** 10.2196/53711

**Published:** 2024-09-26

**Authors:** Sachiko Lim, Paul Johannesson

**Affiliations:** 1 Department of Computer and Systems Sciences Stockholm University Kista Sweden

**Keywords:** infectious disease, ontology, IoT, infectious disease surveillance, patient monitoring, infectious disease management, risk analysis, early warning, data integration, semantic interoperability, public health

## Abstract

**Background:**

Novel surveillance approaches using digital technologies, including the Internet of Things (IoT), have evolved, enhancing traditional infectious disease surveillance systems by enabling real-time detection of outbreaks and reaching a wider population. However, disparate, heterogenous infectious disease surveillance systems often operate in silos due to a lack of interoperability. As a life-changing clinical use case, the COVID-19 pandemic has manifested that a lack of interoperability can severely inhibit public health responses to emerging infectious diseases. Interoperability is thus critical for building a robust ecosystem of infectious disease surveillance and enhancing preparedness for future outbreaks. The primary enabler for semantic interoperability is ontology.

**Objective:**

This study aims to design the IoT-based management of infectious disease ontology (IoT-MIDO) to enhance data sharing and integration of data collected from IoT-driven patient health monitoring, clinical management of individual patients, and disparate heterogeneous infectious disease surveillance.

**Methods:**

The ontology modeling approach was chosen for its semantic richness in knowledge representation, flexibility, ease of extensibility, and capability for knowledge inference and reasoning. The IoT-MIDO was developed using the basic formal ontology (BFO) as the top-level ontology. We reused the classes from existing BFO-based ontologies as much as possible to maximize the interoperability with other BFO-based ontologies and databases that rely on them. We formulated the competency questions as requirements for the ontology to achieve the intended goals.

**Results:**

We designed an ontology to integrate data from heterogeneous sources, including IoT-driven patient monitoring, clinical management of individual patients, and infectious disease surveillance systems. This integration aims to facilitate the collaboration between clinical care and public health domains. We also demonstrate five use cases using the simplified ontological models to show the potential applications of IoT-MIDO: (1) IoT-driven patient monitoring, risk assessment, early warning, and risk management; (2) clinical management of patients with infectious diseases; (3) epidemic risk analysis for timely response at the public health level; (4) infectious disease surveillance; and (5) transforming patient information into surveillance information.

**Conclusions:**

The development of the IoT-MIDO was driven by competency questions. Being able to answer all the formulated competency questions, we successfully demonstrated that our ontology has the potential to facilitate data sharing and integration for orchestrating IoT-driven patient health monitoring in the context of an infectious disease epidemic, clinical patient management, infectious disease surveillance, and epidemic risk analysis. The novelty and uniqueness of the ontology lie in building a bridge to link IoT-based individual patient monitoring and early warning based on patient risk assessment to infectious disease epidemic surveillance at the public health level. The ontology can also serve as a starting point to enable potential decision support systems, providing actionable insights to support public health organizations and practitioners in making informed decisions in a timely manner.

## Introduction

Overall global health has improved over the past 30 years with the steady decline of age-standardized disability-adjusted life-year rates [1]. Although the burden of infectious diseases remains high among children younger than 10 years, the global disease trend has seen a shift from communicable to noncommunicable diseases [1]. Nevertheless, (re)emerging infectious diseases remain global health threats due to climate change, increased wildlife-livestock-human interface associated with urbanization, globalization of transport, and human movement [2]. As we all have witnessed during the COVID-19 pandemic, the rapid and transnational spread of (re)emerging infectious diseases can have catastrophic consequences, including a significant loss of human lives, social disruption, and economic instability.

The COVID-19 pandemic has manifested a global vulnerability to newly emerging infectious diseases. Insufficient epidemic preparedness and delayed responses have exacerbated the spread of SARS-CoV-2 and triggered a significant increase in incidence and mortality. In Italy, for example, it has been claimed that the delayed implementation of the lockdown accounted for a substantial proportion of hospital admissions and deaths [3]. According to a study, if lockdown had been implemented 1 week earlier, Italy could have averted 60% of cases, 48% of intensive care unit admissions, and 44% of deaths at the time of the study [3]. Similarly, another study indicates that if control measures had been implemented just 1-2 weeks earlier, the United States could have avoided 56.5% of reported cases and 54.0% of reported deaths at the time of the study [4].

Moreover, many countries implemented response measures when the local health systems were already becoming overstretched [5]. The prolonged period of health system disruption potentially caused increases in the incidence and mortality of other diseases due to core health service disruptions [6,7]. Thus, early detection is crucial for preventing and responding to emerging infectious disease outbreaks [8]. It demands robust public health surveillance systems to inform effective outbreak management [8]. The main goals of the surveillance are (1) understanding the disease burden and epidemiology; (2) monitoring disease trends; (3) identifying and early warning of public health threats (eg, epidemics of emerging infectious diseases); (4) assessing risks and prioritizing diseases; (5) disseminating surveillance data to stakeholders; and (6) planning, implementing, monitoring, and evaluating public health response measures for disease control, elimination, and eradication.

Traditional infectious disease surveillance is often divided into active and passive surveillance. In active surveillance systems, health department staff proactively contact physicians, laboratories, health care providers, or the general population to collect information about diseases [9]. In passive surveillance systems, which are the most common type of surveillance, medical professionals report cases and deaths to the public health agency according to a list of reportable diseases [9]. While active surveillance is likely to provide complete and more accurate data than passive surveillance, the method is more expensive and labor-intensive. On the other hand, passive surveillance is incomplete and subject to underreporting and delays between event occurrences and notifications [9]. Another major limitation of traditional surveillance systems is that they cannot detect an outbreak in real time.

With advancements in information technologies and the digital revolution, novel surveillance approaches driven by Internet of Things (IoT) have evolved, enhancing traditional infectious disease surveillance systems by enabling real-time detection of outbreaks and reaching a wider population [10]. The IoT creates an ecosystem that connects people and objects through the internet, allowing them to collect and transmit data over a highly distributed network via embedded sensors. The IoT has opened new opportunities to improve public health through enhancing disease surveillance and assisting health care in transitioning to a proactive P4 (predictive, preventive, personalized, and participatory) medicine [10,11]. Innovative IoT-based methods, such as participatory surveillance and digital surveillance, have been used in disease surveillance.

Participatory surveillance that leverages digital connectivity (eg, mobile phone–based apps) requires the direct involvement of system users who voluntarily provide the information needed for informing public health actions. The data collected from each user are aggregated and analyzed for public health purposes. Although there are event-based participatory surveillance systems, many have been used to perform syndromic surveillance. The approach aims to monitor disease indicators in (near) real time for earlier detection of and response to outbreaks to reduce morbidity and mortality [12,13].

The primary advantages of participatory surveillance systems are fourfold [14]: (1) enable large-scale and population-based monitoring at a low cost; (2) enable engagement with populations that are hard to reach by traditional surveillance systems due to geographical constraints or social and economic situations; (3) allow for the rapid 2-way communication between health authorities and system users for public health messaging and education to promote disease prevention and control activities; and (4) provide flexible data systems and user interfaces, which allows health authorities to modify data elements to be collected (eg, adding new symptoms of an emerging infectious disease) and disseminates information in near real time. The app-based technology reduced delays in contact tracing and demonstrated the potential for preventing up to 80% of all transmissions [15]. On the other hand, a significant challenge is recruiting and retaining a representative sample of an at-risk population [14]. The approach also lacks the specificity of a laboratory test to confirm a pathogen, while it can achieve high sensitivity if the surveillance coverage is sufficiently high [14].

Digital public health surveillance uses publicly available user-contributed data collected outside conventional public health surveillance channels. Thus, such data are not generated primarily for infectious disease surveillance [16,17].

As mentioned in the descriptions of various infectious disease surveillance systems, each system has its strengths and limitations, and they complement each other. A hybrid system integrating traditional and novel surveillance approaches is likely to be the most promising option in the big data era [18]. Informing and coordinating effective and timely outbreak management require seamless information flows between disparate heterogeneous surveillance systems. However, they often operate in silos due to a lack of interoperability. Interoperability is defined as “the ability of disparate computer systems or software to exchange data in an efficient and meaningful way [19].” As a life-changing clinical use case, the COVID-19 pandemic has manifested that a lack of interoperability can severely inhibit public health responses to emerging infectious diseases [19]. Interoperability is thus critical for building a robust ecosystem of infectious disease surveillance and enhancing preparedness for future outbreaks.

According to the Healthcare Information and Management Systems Society, interoperability consists of 4 levels: functional (level 1), structural (level 2), semantic (level 3), and organizational (level 4) [20]. This study focuses on the semantic level of interoperability (aka semantic interoperability) to aim for seamless data sharing and integration in an IoT-enhanced surveillance ecosystem that includes various heterogeneous data sources. To achieve semantic interoperability, both data and their unambiguous and shared meaning need to be conveyed to the receiving systems such that they interpret and process the data correctly [21].

In health care settings, reference terminologies or terminology standards have played a significant role in facilitating data standardization and providing semantic interoperability. *ICD* (*International Classification of Diseases*) is the global terminology standard designed to promote international comparability in classifying diseases, injuries, and causes of death. *ICD* is also used to standardize the reporting and monitoring of health conditions [22]. The Systematized Nomenclature of Medicine Clinical Terms (SNOMED CT) is an international clinical reference terminology for facilitating the electronic exchange of clinical health information consistently. SNOMED CT provides the ability to create compositional concepts that combine multiple concepts to form a more detailed representation of a clinical problem statement [23]. It can also be mapped to external coding systems such as *ICD-10* (*International Statistical Classification of Diseases, Tenth Revision*) to promote semantic interoperability. In nursing, NANDA International, the Nursing Interventions Classification, and the Nursing Outcomes Classification are 3 major terminologies that have been used to describe nursing judgments, treatments, and nursing-sensitive patient outcomes [21]. The International Classification for Nursing Practice has also been developed to represent the dynamic nature of nursing practices and their cultural variations [24].

Moreover, various technical interoperability standards have been developed. For example, the HL7/FHIR (Health Level 7 Fast Healthcare Interoperability Resources) is a medical information standard created by HL7 to enable RESTful data exchange [25]. The Observational Medical Outcomes Partnership Common Data Model (OMOP CDM) has been designed to standardize the structure and content of observational data [26]. Clinical Data Interchange Standards Consortium Foundational Standards have been developed to support nonclinical and the life cycle of the clinical research process from planning, data collection, exchange, management, and analysis to reporting of the findings derived from clinical trials [27]. Although various robust reference terminologies and standards are available in clinical medicine, further coordination across standards is necessary to avoid creating standard-specific silos [28].

Another enabler for semantic interoperability is ontology, which refers to “a formal, explicit specification of a shared conceptualization [29].” Ontologies are machine-interpretable and provide the ability to reconcile the meaning of data held across heterogeneous data sources. Data senders and receivers share a common understanding of the meanings of the data exchanged [30,31]. The main strengths of using an ontology are that it provides flexible and technology-agnostic methods for data sharing and integration, expresses relationships between concepts, and enables reasoning [30]. Ontologies are similar to reference terminologies in that they both systematically represent a domain of interest. However, ontologies are more expressive than terminologies, providing richer semantic relationships by representing concepts, their relationships, and axioms [32]. They, thus, serve as a basis for knowledge graphs and also support semantic reasonings.

Enormous efforts have been devoted to developing ontologies to enable data sharing, integration, and analysis for infectious disease surveillance and response. Infectious Disease Ontology (IDO) Core, which was released in 2010, is based on basic formal ontology (BFO) and covers entities and relations relevant to infectious diseases in general. It also includes terms for population-level processes (eg, infection incidence, epidemic, and pandemic) [33]. IDO Core serves as a hub from which extensions based on pathogen type (ie, virus, bacteria, fungi, and parasite) are developed: IDO Virus (VIDO), IDO Bacteria, IDO Fungus, and IDO Parasite [33]. Each of those extensions is further partitioned into pathogen-specific ontologies such as IDO-Dengue Fever (IDODEN) [34], IDO-HIV, and IDO-influenza. To facilitate sharing, integrating, and analyzing COVID-19 data, 3 new IDO extensions have recently been developed [33], namely, VIDO [35], the Coronavirus Infectious Disease Ontology (CIDO) [36] and IDO-COVID-19, which is an extension of CIDO [33]. The Apollo Structured Vocabulary, which is also based on BFO, provides a standardized representation for configurations and output of epidemic simulators [37]. It aims to aid in locating and accessing a simulator, understanding its characteristics, performing analyses, and analyzing outputs to inform policy or decisions about disease control.

To our knowledge, however, no ontology exists that supports IoT-enhanced infectious disease surveillance, risk analysis, and early warning of infectious diseases at individual and public health levels. The aim of this paper is to design an infectious disease ontology that can support data sharing and integration of data collected from IoT-driven patient health monitoring, clinical management of individual patients, and disparate heterogeneous infectious disease surveillance.

The envisaged ontology, called “IoT-based management of infectious disease ontology” (IoT-MIDO), may aid prompt, timely, and concerted responses to infectious disease outbreaks with the effective allocation of limited resources. The novelty and uniqueness of the ontology lies in incorporating IoT-related concepts and concepts relevant to infectious disease surveillance and management. This will facilitate semantic interoperability between IoT-based individual patient monitoring and infectious disease management at the public health level. It could thus ease barriers to bringing benefits to individual as well as population health, which are often seen in isolation due to the historical dichotomization of clinical medicine and public health.

## Methods

### Objectives of the Ontology

We designed an ontology to enhance the collaboration between IoT-driven patient health monitoring, clinical management of individual patients, and infectious disease surveillance. The overall goals of the ontology are to enable the sharing and integration of data collected from disparate heterogeneous surveillance systems and to support risk analysis and early warning for better patient management and triage as well as for early response to infectious disease epidemics. As requirements for the ontology to achieve the intended goals, we formulated the competency questions (CQs) described in Table 1. CQs specify functional requirements for an ontology and are used to evaluate whether the ontology fulfills the elicited requirements [38]. The use of CQs has been proposed in several ontology engineering methodologies such as the Tropos methodology [39] and the NeOn Methodology framework [40].

**Table 1:** Competency questions to elicit and evaluate requirements for IoT^a^-based management of infectious disease ontology.

			CQs^b^
**IoT-driven patient monitoring, risk assessment, early warning, and patient management**			
	CQ1	What information is gathered that can be used for early warning of patients’ health risks?	
	CQ2	Which measurements are monitored using IoT devices?	
	CQ3	What are the main types of recommendations for patient management that can be used for patient triage according to the detected events (ie, anomalies in early warning score)?	
	CQ4	How is it possible to perform contact-tracing activity based on patient information?	
**Clinical management of infectious diseases**			
	CQ5	What is the outcome of an infectious disease process?	
	CQ6	Which treatment provides prophylaxis for the patient?	
	CQ7	Which treatment is used for the disease process?	
	CQ8	Which vaccine is used to mitigate the disease process?	
	CQ9	Which symptoms does a person playing the role of a symptomatic infectious agent carrier develop?	
	CQ10	Which laboratory tests are used for diagnosing a patient?	
	CQ11	Which risk factors can increase the risk of contracting an infectious disease?	
**Epidemic risk analysis**			
	CQ12	What is the risk score for infectious disease epidemic (ie, a score to predict epidemic severity) and the associated risk level?	
	CQ13	What infectious disease control strategies has a country implemented?	
	CQ14	What is the strictness of a country’s response to an infectious disease epidemic?	
**Infectious disease surveillance**			
	CQ15	On which infectious disease does a country conduct surveillance?	
	CQ16	What is an epidemic threshold to determine an infectious disease epidemic?	
	CQ17	What testing strategy does a country have?	
	CQ18	What types of disease surveillance data are collected for infectious disease surveillance?	
	CQ19	What kind of population-based statistics are computed?	
	CQ20	For which infectious diseases is the contact-tracing activity performed?	
	CQ21	To which surveillance system are disease surveillance data reported?	
	CQ22	What is a case definition (ie, a set of standard criteria for identifying cases to monitor the trend of the infectious disease under investigation) of an infectious disease?	
**Transforming individual patient information into surveillance information**			
	CQ23	What patient information is integrated into case-based surveillance data?	

^a^IoT: Internet of Things.

^b^CQ: competency question.

### Design of the Ontology

The IoT-MIDO was developed using the BFO as the top-level ontology. BFO is a domain-independent upper-level ontology created to provide a common top-level structure for enhancing semantic interoperability across different domain ontologies [41]. This facilitates information sharing with multiple ontologies, built upon the BFO by making data smarter by adding both computer and human-interpretable semantics to the raw data. Moreover, when developing the IoT-MIDO ontology, we reused the classes from existing BFO-based ontologies as much as possible to maximize interoperability with other BFO-based ontologies and databases that rely on them. Instead of introducing all of the classes and their definitions and properties, we demonstrate 5 use cases in the results section to show the potential usage of IoT-MIDO, using the simplified ontological models in a similar way to that done by the authors of IDODEN [34]. The complete ontology model and the definitions of all the classes are shown in ﷟Multimedia Appendices 1 and 2.

Table 2 shows the lists of ontology prefix classes that we reused in the use cases. When describing the following use cases, classes are written in italics starting with an upper case (eg, *IoTStream*), and associations are written in italics starting with a lower case (eg, *generatedBy*).

**Table 2:** List of ontology prefixes in which classes are reused and imported into use cases.

Ontology prefix	Ontology full name
FOAF	The Friend Of A Friend ontology [42]
IDOMAL	Malaria Ontology [43]
IoT-Stream	A Lightweight Ontology for IoT (Internet of Things) Data Streams [44]
NCIT	NCI Thesaurus OBO Edition [45]
SOSA	The Sensor, Observation, Sample, and Actuator ontology [46]
UO	Units of Measurement Ontology [47]
geo	GeoSPARQL Ontology [48]
CODO	The COviD-19 Ontology for cases and patient information [49]
EFO	Experimental Factor Ontology [50]
IDO	Infectious Disease Ontology [51]
SYMP	Symptom Ontology [52]
LABO	clinical LABoratory Ontology [53]
OGMS	Ontology for General Medical Science [54]
VO	Vaccine Ontology [55]
ICDO	International Classification of Disease Ontology [56]
GENEPIO	Genomic Epidemiology Ontology [57]
APOLLO_SV	Apollo Structured Vocabulary [37]
TRANS	Pathogen Transmission Ontology [58]
OBI	Ontology for Biomedical Investigations [59]
IDOBRU	Brucellosis Ontology [60]
IoT4PHM	IoT for Patient Health Monitoring Ontology [61]

### Ethical Considerations

This study did not collect any primary or secondary data. Therefore, it was not necessary to obtain approval from the institutional review board. The authors, however, recognize the importance of responsible data handling. Furthermore, informed consent was not applicable, as the study did not directly involve human subjects.

### Disclosure of Generative Artificial Intelligence Usage

We used generative artificial intelligence only for checking and improving formulations. We did not use it for creating the content of the study, including the ontology and use cases.

## Results

### Use Case 1: IoT-Driven Patient Monitoring, Risk Assessment, Early Warning, and Risk Management

**Figure 1:**
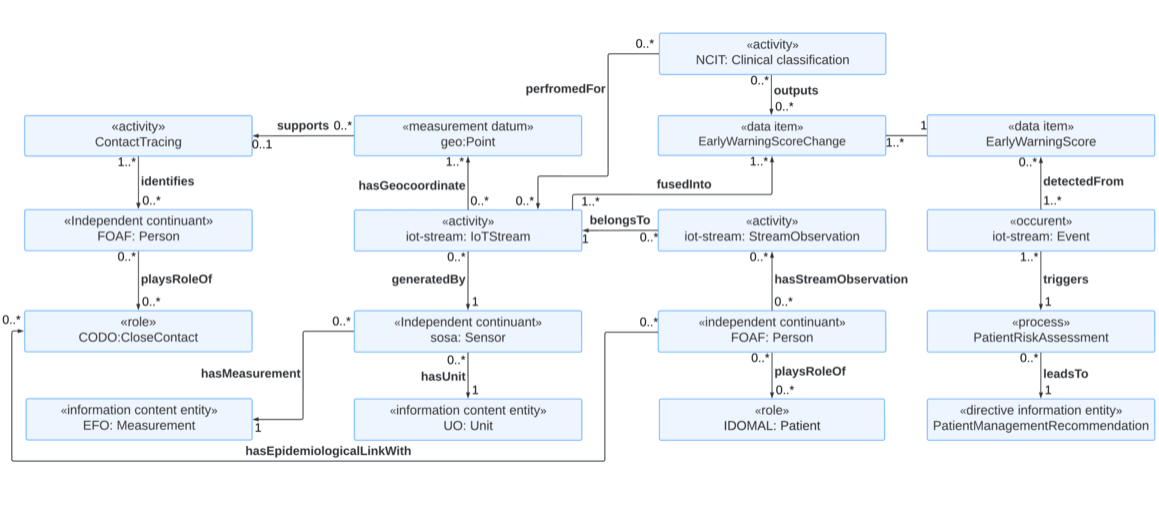
An ontology model for Internet of Things–powered remote monitoring of a patient’s vital signs, health risk assessment, early warning, and proactive health risk management. CODO: The COviD-19 Ontology for cases and patient information; EFO: Experimental Factor Ontology; FOAF: The Friend Of A Friend ontology; IDOMAL: Malaria Ontology; NCIT: NCI Thesaurus OBO Edition; UO: Units of Measurement Ontology.

The first use case is remote monitoring of patients’ vital signs and other health information using IoT devices to detect their health risks proactively (Figure 1). We reused our IoT for patient health monitoring (IoT4PHM) ontology with modifications to create IoT-MIDO [61]. The IoT4PHM was built on the IoT-Stream ontology, consisting of 4 original concepts: IoTStream, StreamObservation, Analytics, and Event. In addition, the ontology is linked with 6 concepts imported from external ontologies: qoi:Quality, iot-lite:Service, sosa:Sensor, qu:QuanityKind, qu:Unit, and geo:Point. We developed the IoT4PHM by adding 3 classes to the IoT-Stream ontology: Patient, UnderlyingHealthcondition, and PatientManagement. The goal of the ontology was to facilitate data integration and sharing, knowledge representation, reasoning, and computer-assisted data analysis to enable IoT-based patient health monitoring and management for the prevention, early detection, and mitigation of patient deterioration. We first modified the IoT4PHM ontology to better conform to BFO by importing classes from BFO-based ontologies (eg, replacing the qu:QuantityKind class with the Measurement class from Experimental Factor Ontology).

### Use Case 2: Clinical Patient Management of Infectious Diseases

**Figure 2:**
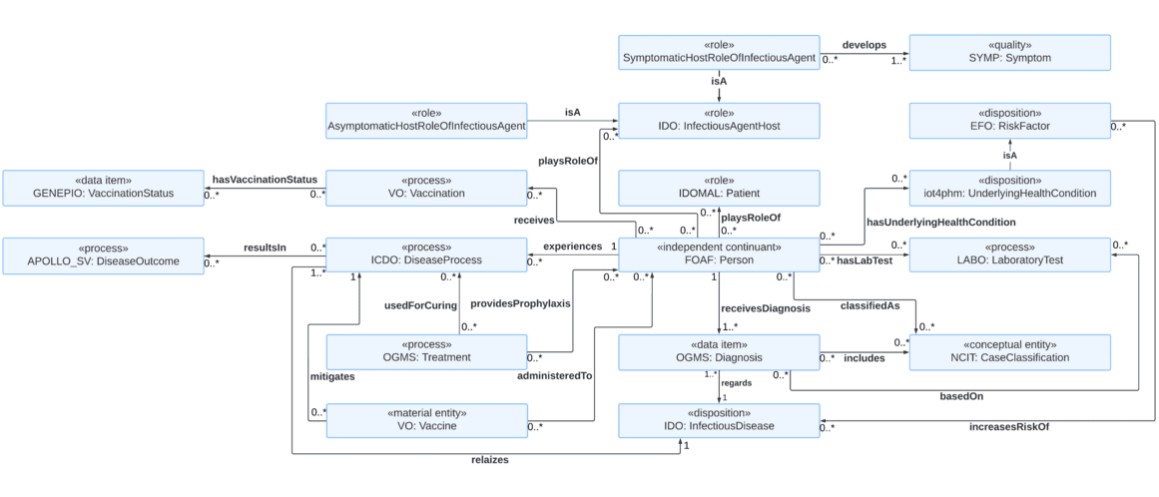
An ontology model for clinical management of infectious diseases in health care settings, supporting the seamless integration of data on vaccination status, laboratory test results, diagnosis, treatment, and underlying health conditions that may affect the course and outcome of an infectious disease. EFO: Experimental Factor Ontology; FOAF: The Friend Of A Friend ontology; GENEPIO: Genomic Epidemiology Ontology; ICDO: International Classification of Disease Ontology; IDO: Infectious Disease Ontology; IDOMAL: Malaria Ontology; LABO: clinical LABoratory Ontology; NCIT: NCI Thesaurus OBO Edition; OGMS: Ontology for General Medical Science; VO: Vaccine Ontology.

The second use case is clinical patient management of infectious diseases (Figure 2). When a *Person* who has acquired an infection seeks health care services, they start to play the role of a *Patient*. The *Patient* receives a *Diagnosis* of *InfectiousDisease* and is classified into *CaseClassification* imported from the Ontology for General Medical Science [54]. The subclasses of *CaseClassification* are *ConfirmedCase, ProbableCase, SuspectedCase*, and *Negative*. The *ConfirmedCase* further has a subclass of *LaboratoryConfirmedCase*, a case confirmed by 1 or more of the laboratory methods that conform to the laboratory criteria included in the case definition. The *Diagnosis* includes information on *CaseClassification*. The latter only specifies whether a person has a particular infectious disease, while *Diagnosis* is a more comprehensive summary of patients’ medical conditions. The *Diagnosis* is based on the *LaboratoryTes*t imported from the clinical LABoratory Ontology [53]. The class includes information on the type of laboratory test the *Patient* has undergone.

An example of a laboratory test is a nucleic acid amplification test, such as a reverse transcription-polymerase chain reaction, an antigen test, and an antibody test in the case of testing for SARS-CoV-2. When a *Person* is identified as a case of a particular infectious disease, they start to play the role of *InfectiousAgentHost,* which has 2 child classes: *AsymptomaticHostRoleOfInfectiousAgent* and *SymptomaticHostRoleOfInfectiousAgent*. In some infections such as SARS-CoV-2, a patient who never has symptoms associated with an infection (ie, an asymptomatic patient) can still transmit an infectious agent to others. The *Person* who plays the role of *SymptomaticHostRoleOfInfectiousAgent* has the *developes* association with the *Symptom*.

The *Patient* experiences the *DiseaseProcess,* which realizes the *InfectiousDisease*. The *Vaccine* administered to the *Person* may mitigate the *DiseaseProcess*. The *Treatment* may either *provideProphylaxis* to the *Person* or is *usedForCuring* the *DiseaseProcess* that the *Person* experiences. The *DiseaseProcess* has the *resultsIn* association with the *DiseaseOutcome* such as death, convalescence, or long-term sequelae. The *Person* receives *Vaccination* using the *Vaccine*, which results in having *VaccinationStatus*.

### Use Case 3: Epidemic Risk Analysis for Timely Response at the Public Health Level

**Figure 3:**
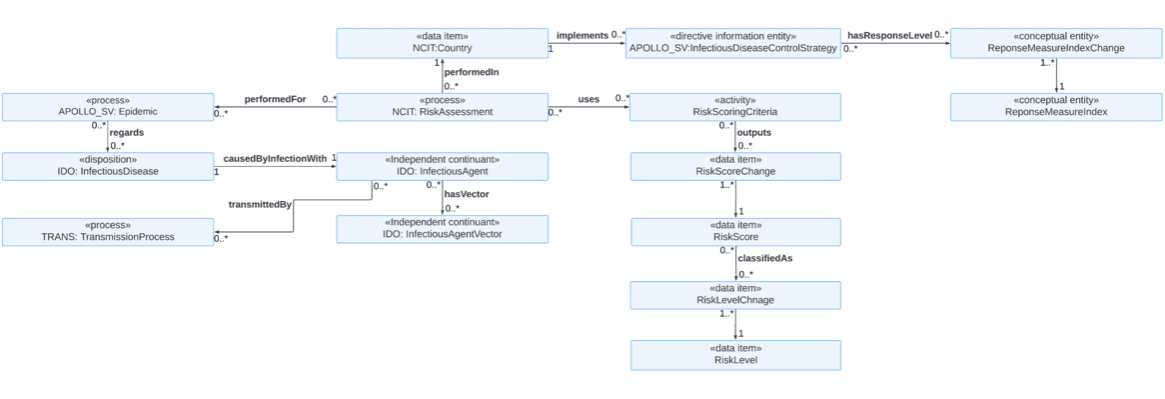
An ontology model for autonomously assessing infectious disease epidemic risk at the national level, with the goal of improving preparedness and promoting timely response. IDO: Infectious Disease Ontology; NCIT: NCI Thesaurus OBO Edition.

The third use case is epidemic risk assessment at the public health level (Figure 3). Risk assessment is essential for informing evidence-based public health decision-making about preparedness for and response to an infectious disease epidemic [62]. We thus added the *RiskAssessment* to handle information on risk assessment for the *Epidemic* regarding the *InfectiousDisease*. The information may include geographical scope (ie, national, subnational, and local or community level), the time of the risk assessment performed, population group (eg, general population and vulnerable population), and the person or the organization that has performed the risk assessment. Various risk-scoring criteria can be used for risk assessment. It is informative to understand which risk-scoring criteria are implemented for risk assessment. The *RiskScoringCriteria* handles information on the criteria’s source, version, and developer. The *RiskScore* represents the overall risk score for an outbreak of an infectious disease in a specific geographical area. Since risk scores may change over time, we also add the *RiskScoreChange* class to track the risk trajectories.

In addition, we included the *RiskLevel* class because risk level (eg, high, moderate, low) is often determined based on the risk score and is frequently used for risk communications (eg, risk assessment reports). Because risk scores can change over time, the risk level also changes accordingly. The *RiskLevelChange* is thus also included. An infectious disease epidemic’s risk depends on the infectious agent’s transmission mode. In our ontology, the *InfectiousAgent* is transmitted by the *TransmissionProcess*. For vector-borne infectious diseases, the *InfectiousAgent* has the *InfectiousVector.* Based on the risk assessment outputs, the *Country* may implement *InfectiousDiseaseControlStrategy*, which has a response level according to the *ResponseMeasureIndex*. The *ResponseMeasureIndexChange* is included to assess the changes in implemented infectious control strategies over time.

**Figure 4:**
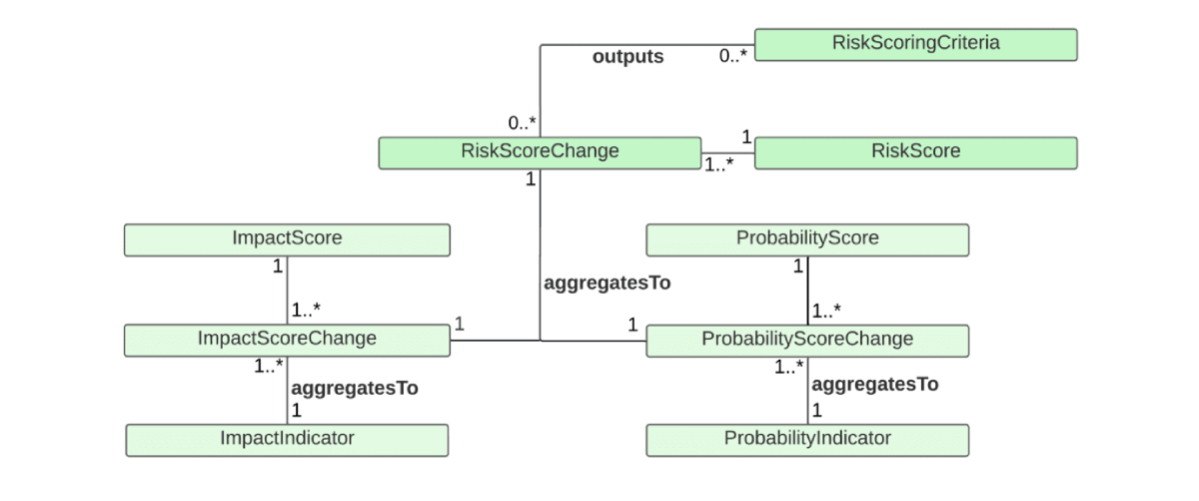
Implementation example using European Centre for Disease Prevention and Control rapid risk assessment methodology [63].

**Figure 5:**
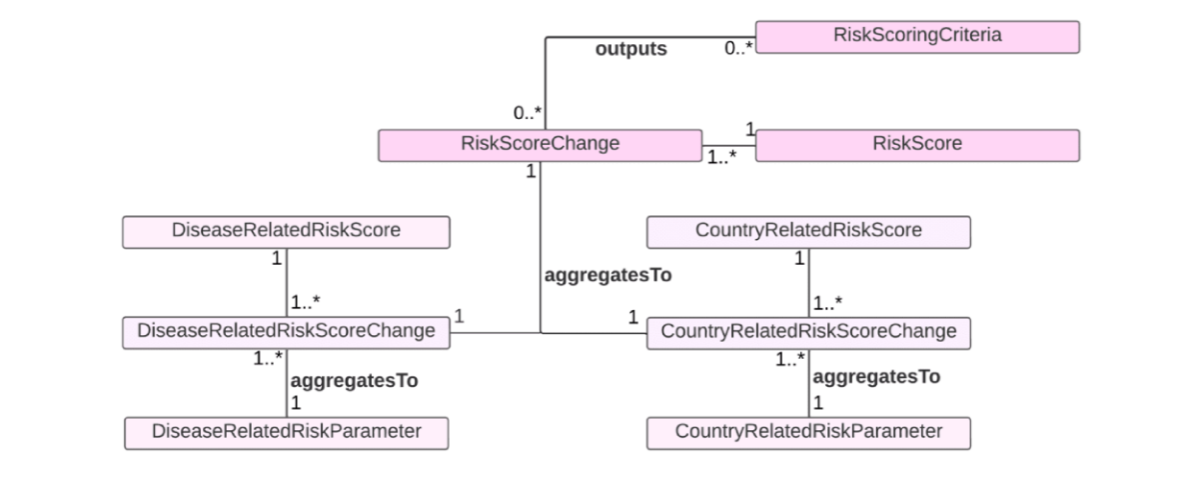
Implementation example of risk assessment based on the proposed framework by Lesmanawati et al [64].

There are various ways of operationalizing risk assessment. For example, one possible way is to use risk score criteria that are proposed by the World Health Organization or the European Centre for Disease Prevention and Control [62,63] (Figure 4 [63]). Both scoring criteria output the overall risk score based on impact score and likelihood or probability scores. For the World Health Organization criteria, the impact is determined by 3 factors: vulnerability assessment, severity assessment, and coping capacity assessment. Another possible way to operationalize is to use the criteria suggested by Lesmanawati et al [64] (Figure 5 [64]). The scoring criteria of their risk analysis framework, called “EpiRisk,” provide rapid risk prediction based on country-specific risk scores computed by summing disease and country risk scores. The disease risk score has 7 parameters (ie, the type of pathogen, basic reproductive number, mode of transmission, the occurrence of asymptomatic transmission, case fatality rate, therapy or drug availability, and vaccine availability). On the other hand, the country risk score has 7 parameters, including the World Bank’s income classification, the proportion of health expenditure to the country’s GDP, the state of peace (assessed by the peace index proposed by the Peace Institute), the type of country border, population density, physician density, and hospital beds per 1000 individuals.

### Use Case 4: Infectious Disease Surveillance

**Figure 6:**
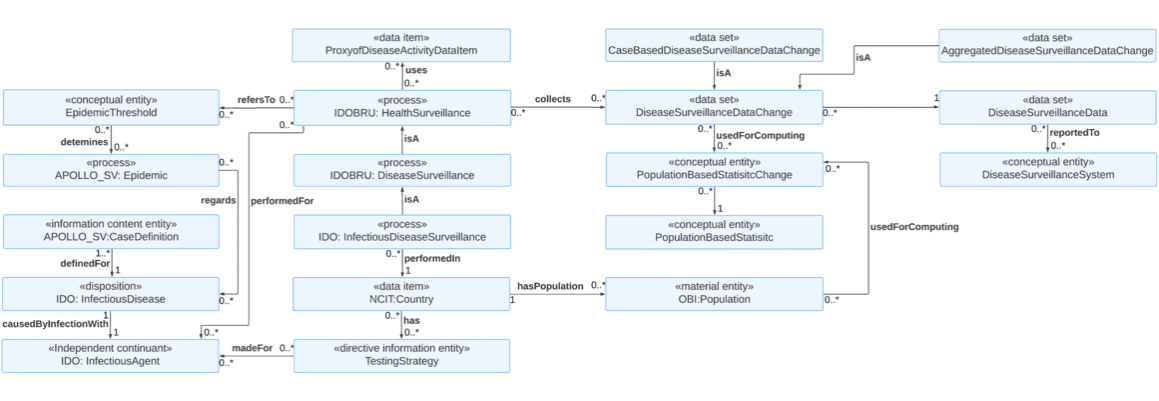
An ontology model for integrating dynamic data on infectious disease case definition, testing strategy, epidemic threshold, and statistics such as morbidity and mortality to track the spread of the disease at the national level. IDO: Infectious Disease Ontology; IDOBRU: Brucellosis Ontology; NCIT: NCI Thesaurus OBO Edition; OBI: Ontology for Biomedical Investigations.

The fourth use case is modeling infectious disease surveillance (Figure 6). The *InfectiousDiseaseSurveillance* is a subclass of *DiseaseSurveillance,* which is a subclass of *HealthSurveillance*. The *HealthSurveillance* is performed for an *InfectiousDiseaseAgent*. The *InfectiousDiseaseSurveillance,* performed in a particular *Country,* collects *DiseaseSurveillanceData* that are reported to the *DiseaseSurveillanceSystem* and are used for computing the *PopulationBasedStatistic* based on the *Population* data of the *Country*. The *DiseaseSurveillanceData* has 2 subclasses: *CaseBasedDiseaseSurveillanceData* and *AggregatedDiseaseSurveillanceData*. We also included *DiseaseSurveillanceDataChange* for handling longitudinal surveillance data.

*HealthSurveillance* refers to the *EpidemicThreshold,* which defines the *Epidemic* regarding *InfectiousDisease*. The *CaseDefinition* is a set of standard criteria for identifying cases to monitor the trend of the *InfectiousDisease* under investigation [65]. Using uniform case definitions is essential for public health surveillance. By ensuring that every case is equivalent, the number of cases and disease incidence across different time points and geographical areas can be meaningfully compared [66]. Case definitions can vary across countries, especially at an early stage of newly emerged infectious disease outbreaks [67]. Furthermore, the definitions can be modified as new evidence on infectious diseases becomes available. When analyzing and interpreting surveillance data, it is essential to know on which case definition the diagnosis of an infectious disease is based. The number of cases needs to be interpreted with caution when a new version becomes available. The *CaseDefinition* may include information on the source of the case definition and its version and has 4 subclasses: laboratory criteria, clinical criteria, epidemiological criteria, and diagnostic imaging criteria.

*HealthSurveillance* can use the *ProxyDiseaseActivityDataItem*, which can include data items that may provide an earlier indication of an epidemic spread than traditional epidemiological metrics such as confirmed cases or deaths [68]. Kogan et al [68] evaluated 6 digital data sources as proxies of COVID-19 activity to detect COVID-19 outbreaks as early as possible [68]:

1. Google Trends patterns for a suite of COVID-19–related terms
2. COVID-19–related Twitter activity
3. COVID-19–related clinician searches from UpToDate
4. Predictions by the global epidemic and mobility model, a state-of-the-art metapopulation mechanistic model
5. Anonymized and aggregated human mobility data from smartphones
6. Kinsa smart thermometer measurements

They found that increased digital data stream activity anticipates the increase in confirmed cases and deaths 2-3 weeks earlier than traditional surveillance methods. Although all metrics discussed in their study have limitations, the authors proposed using the combination of disparate health and behavioral data or early warning of increased COVID-19 activity [68]. We believe that those digital proxy data become an important asset for future infectious disease surveillance; thus, they are included in our ontology.

### Use Case 5: Ontological Model of Transforming Patient Information Into Surveillance Information

**Figure 7:**
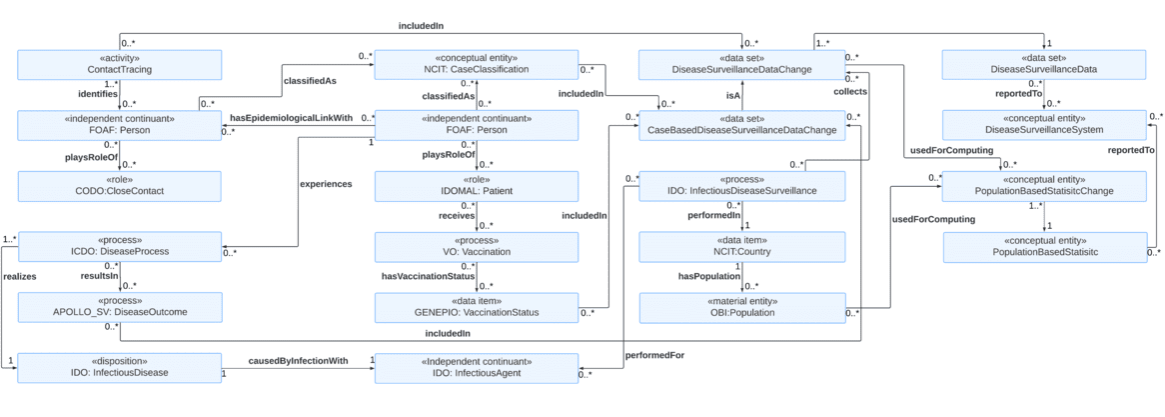
An ontology model for aggregating individual patient data into surveillance information to provide actionable insights for informed decision-making and timely public health interventions. CODO: The COviD-19 Ontology for cases and patient information; FOAF: The Friend Of A Friend ontology; GENEPIO: Genomic Epidemiology Ontology; ICDO: International Classification of Disease Ontology; IDO: Infectious Disease Ontology; IDOMAL: Malaria Ontology; NCIT: NCI Thesaurus OBO Edition; OBI: Ontology for Biomedical Investigations; VO: Vaccine Ontology.

The last use case is transforming patient information into surveillance information (Figure 7). Individual patient information such as *VaccinationStatus*, *CaseClassification*, and *DiseaseOutcome* is included in the *CaseBasedSurveillanceDataChange*. The information on *ContactTracing* may also be included in the *DiseaseSurveillanceDataChange*. This contributes to transforming individual patient information into data sets that can be used for infectious disease surveillance. The rest of the parts in Figure 5 [64] have already been described in the use cases 2 and 4.

### Answers to CQs

#### IoT-Driven Patient Monitoring, Risk Assessment, Early Warning, and Patient Management

The answer to CQ1 is that the *EarlyWarningScore* class handles the information on the absolute value of the early warning score that is computed based on a particular *ClinicalClassification* scheme. An anomaly in the early warning score is detected as an *Event* that warns patients of health risks. A type of measurement that an IoT device is monitoring can be found in the *Measurement* class, which has the subclasses *PhysiologicalMeasurement* and *EnvironmentalMeasurement*. This answers CQ2. The answer to CQ3 is that the *PatientManagementRecommendation* has 3 subclasses: *HealthEducation, ReferralToHealthcar*e, and *EmergencyAlert* depending on the severity of the detected *Event* (eg, an early warning score). The classification can serve as triage which helps ensure the effective allocation of limited resources to those who need it most and prevent overburdening health care systems. The answer to CQ4 is that an *IoTStream* generated by the *Sensor* has a *hasGeocoordinate* relationship with the *Point* class. The information on the geolocation handled in the *Point* class can support the *ContactTracing* activity by using positioning systems.

#### Clinical Management of Infectious Diseases

The answer to CQ5 is that the outcome of an infectious disease process that a *Patient* experiences is handled in the *DiseaseOutcome,* which can have instances of convalescence, death, intensive care unit admission, and hospitalization. The answer to CQ7 and CQ8 is that the *Treatment* class has a *provideProphylaxis* association with the *Patient* class and has the *usedForCuring* association with the *DiseaseProcess*. Thus, the information on medications used for treating an infectious disease and prophylaxis can be handled using our ontology. The answer to CQ8 is that the *Vaccine* having a mitigated association with the *DiseaseProcess* may include information such as the identifier of the vaccine, the status code of the vaccine, the administered vaccine product, the vaccine manufacturer, the expiration date, the administered date, a performer who administered the vaccine to a person, and possible side effects that can be caused by the vaccine. The answer to CQ9 is that the *Symptom* class represents symptoms developed by the symptomatic patients, which is especially important for characterizing newly emerging infectious diseases. The answer to CQ10 is that a *Person* playing the role of the *Patient* has a *hasLabTes*t association with the *LaboratoryTest* class, which has a *basedOn* association with the *Diagnosis*. The *LaboratyTest* represents the information on the laboratory test used for the patient. The *Diagnosis* includes the *CaseClassification* of the *Person,* which includes instances of confirmed case, laboratory-confirmed case, probable case, suspected case, and negative. The answer to CQ11 is that the *UnderlyingHealthCondition* class handles the information on the risk factors that pose an increased risk of an infectious disease. This information is critical to identifying high-risk groups who need to be prioritized for treatment and public health interventions.

#### Epidemic Risk Analysis

The answer to CQ12 is that the *RiskAssessment* class, which is performed for the *InfectiousAgent*, uses the *RiskScoringCriteria* to output the *RiskScore* that can be classified as a *RiskLevel* such as very high, high, medium, low, or very low. The answer to CQ13 is that the *Country* class has the *implements* association with the *InfectiousDiseaseControlStrategy* so that it allows the retrieval of information on how a country responds to an infectious disease epidemic at a given time. This information is important for evaluating the effectiveness of the control strategies in reducing the transmission of an infectious disease. The answer to CQ14 is that the *InfectiousDiseaseControlStrategy* has the *hasResponseLevel* association with the *ResponseMeasureIndex* class, which handles the information on the score showing the strictness of government responses. The *ResponseMeasureIndex* has a subclass of *OxfordCOVID-19GovenmentSringencyIndex*, which systematically collects data related to closure and containment and health and economic policy from more than 180 countries and territories, which allows comparisons of policy responses within and across countries over time [69]. If another stringency index is preferred, a user can add it as a subclass of the *ResponseMeasureIndex* on demand. To track the change in stringency index scores, we introduced the *ResponseMeasureIndexChange* class in the model.

#### Infectious Disease Surveillance

The answer to CQ15 is that *HealthSurveillance*, which is the upper class of *InfectiousDiseaseSurveillance*, has a *performedIn* association with the *Country* and has a *performedFor* association with the *InfectiousAgent*. Thus, it is possible to model multiple infectious disease surveillances for different infectious agents that have been or are currently underway in a particular country. The answer to CQ16 is that *HealthSurveillance* has a *refersTo* association with the *EpidemicThreshold*. The *EpidemicThreshold* has a *determine* association with the *Epidemic* class and has a *thresholdFor* association with the *InfectiousDisease*. For example, crossing the epidemic threshold of 10% positive influenza laboratory tests indicates increased influenza activity and thus the start of the seasonal epidemic [70]. The answer to CQ17 is that *Country* has an association with the *SamplingStrategySpecification,* which has a subclass of *TestingStrategy*. Knowing that case counts are reported under each testing strategy is vital since the changes in testing strategies significantly influence case counts, and thus the epidemic dynamics need to be interpreted with caution when there are changes in testing strategies [71,72]. The answer to CQ18 is that *DiseaseSurveillanceData* has 2 subclasses: *CaseBasedInfectiousDiseaseSurveillanceData* and *AggregatedInfectiousDiseaseData*. Thus, both types of surveillance data can be represented in each subclass since some countries report case-based data while others submit only aggregated data. The answer to CQ19 is that the *PopulationBasedStatistic* can have subclasses as necessary, such as *CaseNotificationRate, DeathRate, HospitalAdmissionRate, HospitalBedOccupancyRate, TestPositivityRate, TestingRate, VaccinationUptake, EffectiveReproductiveNumber, Seroprevalence,* and *CaseFatalityRate*. The *Country* has a *Population,* which has an *useForComputing* association with *PopulationBasedStatistic* since the (sub)population data are necessary for computing population-based statistics. The answer to CQ20 is that *ContactTracing* has a *performedFor* association with the *InfectiousDisease* class. Thus, contact-tracing activities for multiple infectious diseases can be modeled simultaneously. The answer to CQ21 is that the *DiseaseSurveillanceData* has a *reportedTo* association with *DiseaseSurveillanceSystems* since several infectious disease surveillance systems collect different types of disease surveillance data that may be combined for analysis, and knowledge of the provenance of data is thus needed when referencing to the raw data. The answer to CQ22 is that the *CaseDefinition* has a *definedFor* association with the *InfectiousDisease* and has a *basedOn* association with the *CaseClassification*. The case definition is a set of criteria for systematically counting cases and thus is indispensable for infectious disease surveillance. The *CaseDefinition* has 4 subclasses: *LaboratoryCriteria, ClinicalCriteria, EpidemiologicalCriteria, and DiagnosticImagingCriteria*.

#### Transforming Individual Patient Information Into Surveillance Information

The answer to CQ23 is that the *CaseClassification*, the *VaccinationStatu*s, and the *DiseaseOutcome* classes have an *includedIn* association with the *CaseBasedDiseaseSurveillanceData*. Thus, individual patients’ data on case classification, vaccination status, and disease outcomes can be integrated seamlessly into surveillance data through the *CaseBasedDiseaseSurveillanceData* class.

Therefore, the IoT-MIDO successfully addresses all of the CQs and can potentially be used for (1) IoT-driven remote patient health monitoring, risk assessment, early warning, and patient management in the context of infectious disease outbreaks, (2) clinical management of infectious diseases, (3) epidemic risk analysis, (4) infectious disease surveillance, and (5) transforming individual patient information into surveillance information.

## Discussion

### Principal Findings

The aim of our work was to design an infectious disease ontology that can support semantic data integration from disparate heterogeneous sources, including IoT-driven patient monitoring, clinical management, and infectious disease surveillance systems, and interlinking them. For this end, we developed the IoT-MIDO ontology, and by addressing CQs and presenting 5 use cases, we demonstrated the potential of this ontology to enhance data interoperability, integration, and analysis across health care and public health domains, and to assist health care practitioners and public health decision makers in interpreting data available and their semantic relationships. The IoT-MIDO ontology can also serve as a starting point for enabling automated reasoning to drive actionable insights and informed decision-making to improve the health of individual patients as well as populations at large.

### Comparing IoT-MIDO and OMOP CDM

Similar to our work, the OMOP CDM can be used to support data integration from disparate sources in the context of infectious diseases. The model was developed by the Observational Health Data Sciences and Informatics as an open-source common data standard to store observational health data [73]. The OMOP CDM effectively leverages a relational database schema to represent structured tabular data. Through integration with standardized vocabularies, it facilitates data analysis across multiple data sources and meaningfully compares and reproduces results from various observational studies [73].

However, in contrast to OMOP CDM, we have chosen an ontology modeling approach for knowledge representation because of the following capabilities. Ontologies offer more comprehensive and explicit knowledge representation, enriching data with richer semantic contexts and meanings, and modeling relationships between concepts in both human- and machine-interpretable formats [74]. Their expressiveness provides capabilities for automated reasoning, inferencing, and more precise querying over data.

Furthermore, ontologies provide greater flexibility in modeling complex relationships beyond mere hierarchies, accommodating any data formats, including structured, unstructured, and semistructured data [75]. While their flexibility enables more seamless data integration, their ease of extensibility allows for adaptation to the dynamic growth of data.

Ontologies are compatible with the World Wide Web Consortium standards for the Semantic Web. The World Wide Web Consortium endorses the use of International Resource Identifiers [76] and Uniform Resource Identifiers [77] and Uniform Resource Identifiers allows data from distributed and heterogeneous systems and databases to be linked together on the Web of Linked Data. This enables the establishment of semantic links between IoT-MIDO and existing specific infectious disease ontologies such as IDODEN [34] and IDO-COVID-19 [33], addressing the major issue of siloed information across disconnected systems, which prevents a comprehensive understanding of public health data.

### Compatibility With Other BFO-Based Ontologies

There are many well-established BFO-based ontologies existing, such as VIDO [35], CIDO [36] and IDO-COVID-19 [33], which are all ultimately extended from IDO [51]. IoT-MIDO adopts terms from those established ontologies, such as *Infectious disease* and *Infectious agent,* and *infectious disease surveillance* from IDO. This provides the possibility of easily extending IoT-MIDO further, according to the needs of ontology users to create semantic relationships with other BFO-based ontology concepts, for example, to characterize virus species and to overlapping or concurrently occurring multiple infectious disease epidemics.

The novelty and uniqueness characterizing our ontology lie in adopting concepts from IoT-Stream [44], such as *IoT stream* and *stream observation*. Incorporating these concepts, together with concepts related to infectious disease surveillance, control strategy, and response measure, which are both newly created and adopted from existing ontologies, builds a bridge to link IoT-based individual patient monitoring and early warning based on patient risk assessment to infectious disease epidemic surveillance at the public health level.

### Strengths, Limitations, and Future Directions

This study introduced IoT-MIDO, making a crucial initial step in addressing the long-standing issue of information silos caused by the historical segmentation between clinical medicine and public health, as well as the lack of interoperability across disparate systems. We made efforts to minimize the creation of new ontology concepts, opting instead to reuse existing domain-specific ontology concepts, particularly those based on BFO, to enhance semantic interoperability with external ontologies. Furthermore, digital data collected through IoT-driven systems provide information, including patients’ real-time physical and mental health statuses, lifestyles, and sociodemographics [78]. Such data are underutilized in the context of infectious disease surveillance, yet they can enhance existing surveillance systems by integrating with data obtained using conventional surveillance approaches. This integration can contribute to the development of more comprehensive epidemiological profiles at public health level.

However, 2 primary limitations must be acknowledged. First, the ontology has yet to undergo formalization using dedicated ontology development tools, such as Protégé. We recognize this shortfall and intend to rectify it by formalizing the ontology in the Web Ontology Language using Protégé in future iterations. Second, our evaluation of the ontology has been primarily focused on its ability to answer CQs. We acknowledge the need for iterative empirical evaluation using live data sets to assess the capabilities of the ontology to integrate individual patient and surveillance data. Such evaluations are crucial not only for validating its practical use but also for facilitating continuous enhancements to its quality over time.

These limitations highlight areas for future research and development. Moreover, several studies have proposed ontology-driven approach to ensure data compliance [79-81]. For instance, Debruyne et al [79] proposed an ontology, an extension of the provenance ontology called “PROVO,” to represent collected informed consent and its changes over time [79]. While many of existing studies predominantly focus on verifying compliance with policies regulations during the data-processing stage, their ontology model is used to assess potential compliance issues at data set creation stage before the data undergo processing.

Inspired by these studies, we plan to expand our ontology to include entities representing health data security, privacy, informed consent, and compliance with the legal requirements such as General Data Protection Regulation in future versions. Incorporating these entities is essential for safeguarding patients’ sensitive information and ensuring compliance with legal requirements and contractual agreements. Enabling compliance verification at the data set creation stage saves time and resources that would otherwise be spent on post hoc compliance analysis during data processing. In addition, it strengthens data security and privacy measures by identifying and addressing vulnerabilities or compliance gaps early in the data life cycle.

### Conclusions

The primary aim of IoT-MIDO is to address the issue of information silos between clinical medicine and public health and enhance interoperability across different systems. Demonstrating IoT-MIDO’s capability to answer diverse CQs highlights its potential as a tool for achieving this goal. The seamless integration and sharing of clinical and public health data are particularly important during the rise of emerging infectious disease outbreaks when knowledge and evidence about infectious disease agents, transmission routes, and disease profiles are still scarce. This integration can accelerate the understanding of comprehensive and consistent epidemiological profiles and facilitate the effective and timely planning and execution of preventive and control measures.

As health data volume and complexity grow with increasing IoT applications in health care, standardizing such disparate heterogeneous data is essential for the effective and efficient use and integration of such data in infectious disease surveillance. Ontologies such as IoT-MIDO help systematically represent knowledge to make disparate heterogeneous data ready for integration and comparable for further analyses, enabling informed and proactive interventions at both individual and population levels. IoT-MIDO’s interoperability with existing ontologies, especially BFO-based ones like IDO, also facilitates seamless data exchange and the creation of comprehensive surveillance ecosystems.

Moreover, while the ontology standardizes the way of data sharing, it also provides flexibility to meet local needs, for example, by adding country-specific concepts and reference data such as codes while semantically linking them to global concepts or reference data.

Looking ahead, IoT-MIDO could underpin advanced decision support systems and predictive analytics tools in health care. These systems, leveraging computational semantic and logical reasoning, can assist in accurate diagnoses and optimal treatment decisions while enabling proactive surveillance and early detection of public health threats.

IoT-MIDO contributes to using IoT technologies to enhance health care delivery and modernize infectious disease surveillance by providing means to bridge individual patient data with epidemiological data. By enabling the integration of these data from multiple domains, the ontology enhances interoperability, thereby advancing the use of IoT technologies in providing personalized preventive measures and care for individual patients as well as improving preparedness and response to infectious disease epidemics.

## Appendix

Multimedia Appendix 1 The entire model of the IoT-MIDO. IoT-MIDO: Internet of Things–based management of infectious disease ontology.

Multimedia Appendix 2 IoT-MIDO definition table. IoT-MIDO: Internet of Things–based management of infectious disease ontology.

## Abbreviations

BFO: basic formal ontology
CIDO: Coronavirus Infectious Disease Ontology
CQ: competency question
HL7/FHIR: Health Level 7 Fast Healthcare Interoperability Resources
ICD: International Classification of Diseases
ICD-10: International Statistical Classification of Diseases, Tenth Revision
IDO: Infectious Disease Ontology
IDODEN: Infections Disease Ontology–Dengue Fever
IoT: Internet of Things
IoT4PHM: Internet of Things for patient health monitoring
IoT-MIDO: Internet of Things–based management of infectious disease ontology
OMOP CDM: Observational Medical Outcomes Partnership Common Data Model
SNOMED CT: Systematized Nomenclature of Medicine Clinical Terms
VIDO: Virus Infectious Disease Ontology

## Data Availability

The link to the IoT-based management of infectious disease ontology model and additional information, including definitions of the entities and their attributes, is provided in Multimedia Appendices 1 and 2.
