# Supplementary figures and images for: An Ontology to Bridge the Clinical Management of Patients and Public Health Responses for Strengthening Infectious Disease Surveillance: Design Science Study
Source: JMIR Form Res. 2024 Sep 26;8:e53711. doi: 10.2196/53711 (PMC11467600; doi:10.2196/53711)

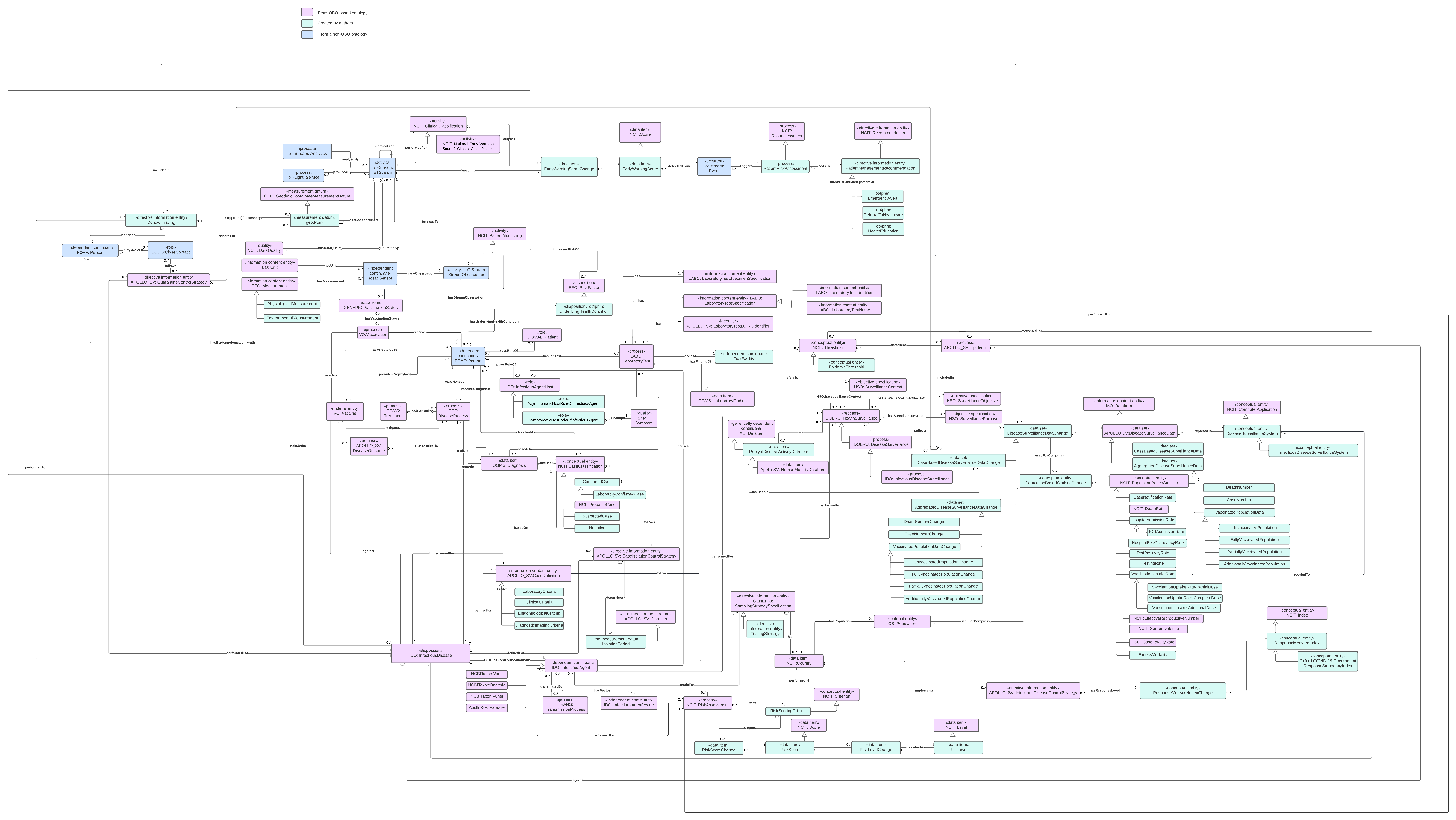

Supplement: Multimedia Appendix 1 [file formative_v8i1e53711_app1.png]
